# Differential expression profiles of long non-coding RNAs as potential biomarkers for the early diagnosis of acute myocardial infarction

**DOI:** 10.18632/oncotarget.20101

**Published:** 2017-08-09

**Authors:** Ling Li, Yingying Cong, Xueqin Gao, Yini Wang, Ping Lin

**Affiliations:** ^1^ Department of Cardiology, The Second Affiliated Hospital of Harbin Medical University, Harbin, China; ^2^ Department of Biological Sciences and Arnie Charbonneau Cancer Institute, University of Calgary, Calgary, Canada; ^3^ Medical Department of Breast Oncology, The Tumor Hospital of Harbin Medical University, Harbin, China; ^4^ School of Nursing, Harbin Medical University, Harbin, China

**Keywords:** acute myocardial infarction, early diagnosis, long non-coding RNAs, expression profiles, biomarkers

## Abstract

Acute myocardial infarction (AMI) is a major cause of morbidity and mortality worldwide. The early diagnosis of AMI is crucial for deciding the course of treatment and saving lives. Long non-coding RNAs (lncRNAs) are recently discovered ncRNA class and their dysregulated expression has been implicated in cardiovascular diseases. In this study, we analyzed lncRNA expression pattern by using two microarray datasets of AMI and healthy samples from the Gene Expression Omnibus (GEO) database and tried to identify novel AMI-related lncRNAs and investigate the predictive roles of lncRNAs in the early diagnosis of AMI. From the discovery cohort, 11 differentially expressed lncRNAs were identified as candidate biomarkers that were validated in the discovery cohort, internal cohort and an independent cohort, respectively. Hierarchical clustering analysis suggested that the expression pattern of these 11 candidate lncRNA biomarkers was closely associated with disease status of samples. Then a lncRNA risk classifier was developed by integrating expression value of 11 differentially expressed lncRNAs using support vector machine (SVM) algorithm. The results of leaving one out cross-validation (LOOCV) suggested that the lncRNA risk classifier has a good discrimination between AMI patients and healthy samples with the area under ROC curve (AUC) of 0.955, 0.92 and 0.701 in three cohorts, respectively. Functional enrichment analysis suggested that these 11 candidate lncRNA biomarkers might be involved in inflammation- and immune-related biological processes. Our study indicates the potential roles in the early diagnosis of AMI and will improve our understanding of the molecular mechanism of the occurrence and recurrence of AMI.

## INTRODUCTION

Acute myocardial infarction(AMI), commonly known as aheart attack, is a major cause of morbidity and mortality worldwide. It is estimated that there was about 15.9 million MI occurred in 2015 [1]. The early diagnosis of AMI is crucial for deciding the course of treatment and saving lives, because the highest risk of fatality occurs within the initial hours of onset of AMI [2]. The conventional method is based on physical examination together with electrocardiogram and the measurement of gold standard cardiac biomarkers [3], but they suffer from a lack of high specificity and sensitivity. Therefore, the identification of new biomarkers in early diagnosis of AMI remains to be needed.

Recent advances in the sequencing and analysis of the human genome have led to the discovery of thousands of previously unannotated non-coding transcripts, including small non-coding RNAs and long non-coding RNAs (lncRNAs) [4]. lncRNAs, a recently discovered ncRNA class, are defined as non-protein coding transcripts longer than 200 nucleotides which arbitrarily distinguish from small non-coding RNAs [5]. Increasing evidence suggested that lncRNAs play various biological roles including epigenetic gene regulation, transcriptional gene regulation, posttranscriptional gene regulation by function as signals, decoys, scaffolds and guides [6]. lncRNAs are emerging as important regulators of tissue physiology and disease processes, and the dysregulation of lncRNAs has been implicated in many human complex diseases [7–9]. Moreover, lncRNA expression can be measured in urine and blood, making them attractive biomarkers of diagnosis and prognosis for diseases [10]. Recent studies have reported the link between cardiovascular diseases and lncRNAs, including AMI. Some lncRNAs were identified to be significantly and differentially expressed between AMI and healthy samples in different studies [11–13]. However, the utility of lncRNAs as biomarkers for early diagnosis of AMI needed to be further investigated.

In this study, we aimed at exploring the potential of lncRNAs as biomarkers for early diagnosis of AMI and tried to identify lncRNA biomarkers associated with AMI by analyzing genome-wide lncRNA expression profiles in the discovery cohort and confirming the diagnostic value of the identified lncRNA biomarkers in the internal validation cohort and an independent cohort from Gene Expression Omnibus (GEO) database. Besides, we further examined whether there dysregulated lncRNA expression is pervasive in AMI recurrence, and investigated the functional implication of identified lncRNA biomarkers in AMI.

## RESULTS

### Identification of differentially expressed lncRNAs between AMI patients and healthy samples

**Figure 1:**
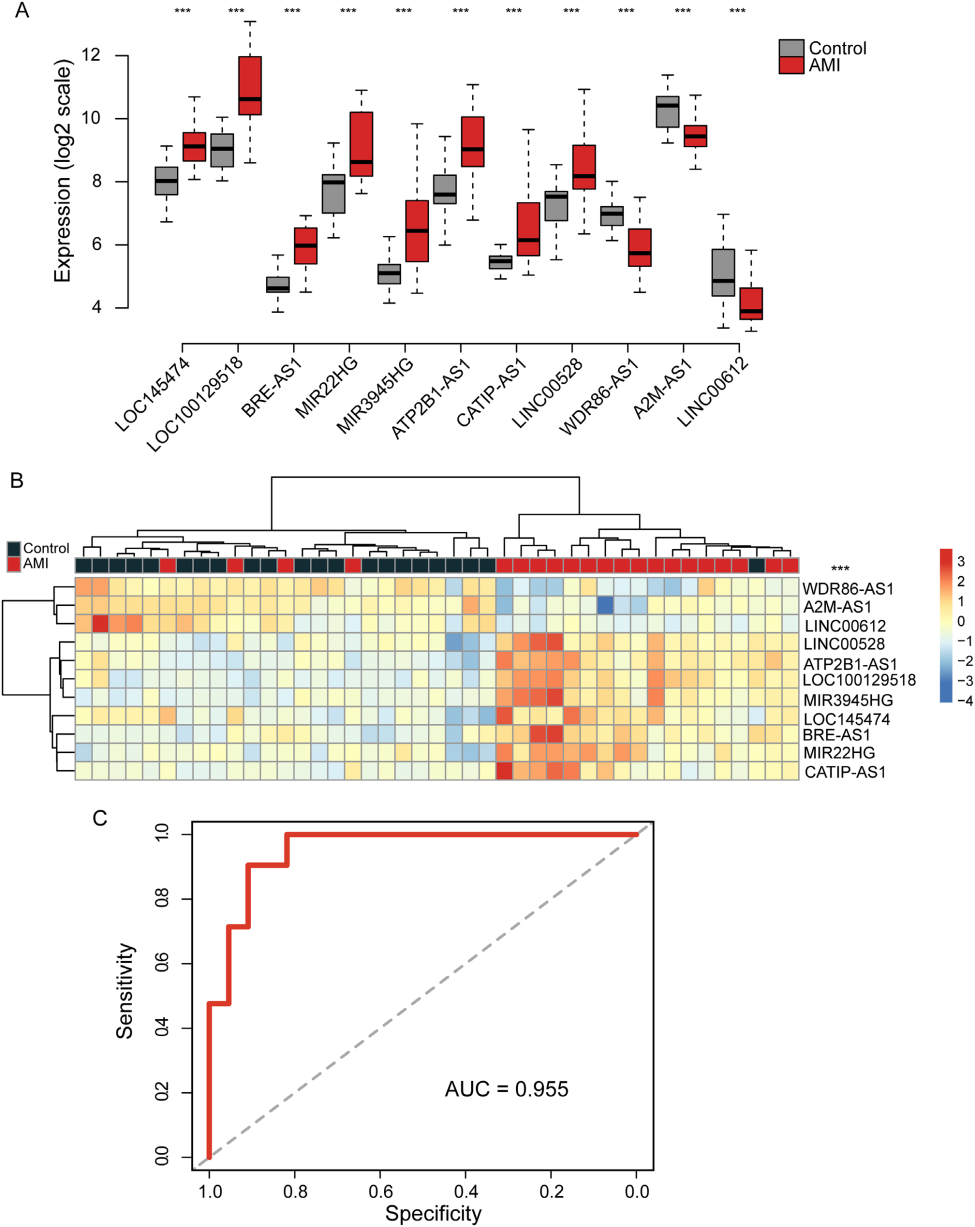
Identification of differentially expressed lncRNAs between AMI patients and healthy samples in the discovery cohort **(A)** Expression distribution of 11 differentially expressed lncRNAs in AMI patients and healthy samples in the discovery cohort measured by microarray. **(B)** The heatmap of hierarchical clustering of differentially expressed lncRNAs for all samples in the discovery cohort. **(C)** Receiver operating characteristic curves of SVM-based lncRNA risk classifier in the discovery cohort.

We first compared the lncRNA expression profiles between 21 AMI patients and 22 healthy samples in the discovery cohort and performed significant analysis of microarray (SAM). Finally, a total of 11 lncRNAs with an adjusted P-value <0.05 after Benjamini & Hochberg correction and fold change > 2.0 (<0.5) was found to have significant differential expression pattern in AMI patients as compared to healthy samples (Supplementary Table 1). Of these, 8 lncRNAs were over-expressed including *LOC145474*, *LOC100129518*, *BRE-AS1*, *MIR22HG*, *MIR3945HG*, *ATP2B1-AS1*, *CATIP-AS1* and *LINC00528*, and 3 lncRNAs were down-regulated, including *WDR86-AS1*, *A2M-AS1* and *LINC00612*. The distribution of expression levels of 11 differentially expressed lncRNAs was shown in Figure 1A.

### Diagnostic value of differentially expressed lncRNAs for AMI

To test the predictive value of these 11 differentially expressed lncRNAs or the early diagnosis of AMI, we first performed hierarchical clustering analysis for all samples in the discovery cohort according to the 11 differentially expressed lncRNAs. As shown in Figure 1B, two distinctive sample groups were obtained by hierarchical clustering analysis: the first sample group contained 25 samples (21 healthy samples and 1 AMI patients) and the second sample group contained 18 samples (1 AMI patients and 17 healthy samples) achieving a prediction accuracy of 95.4%. The disease status of the two sample groups was significantly different (p<0.001, chi-square test). Then we further examined the diagnostic value of these 11 differentially expressed lncRNAs in AMI using support vector machine (SVM) with the sigmoid kernel method and leave one out cross-validation (LOOCV) strategy. A lncRNA risk classifier was developed by integrating expression value of 11 differentially expressed lncRNAs using SVM method. The results of LOOCV suggested that the lncRNA risk classifier has a good discrimination between AMI patients and healthy samples with the area under ROC curve (AUC) of 0.955 (Figure 1C), the sensitivity of 71.4% and specificity of 90.9%. The DOR was 25. These results showed that 11 differentially expressed lncRNAs had a diagnostic value in AMI patient and may be used as candidate biomarkers for the early diagnosis of AMI.

### Validation of candidate lncRNA biomarkers in the internal validation cohort

**Figure 2:**
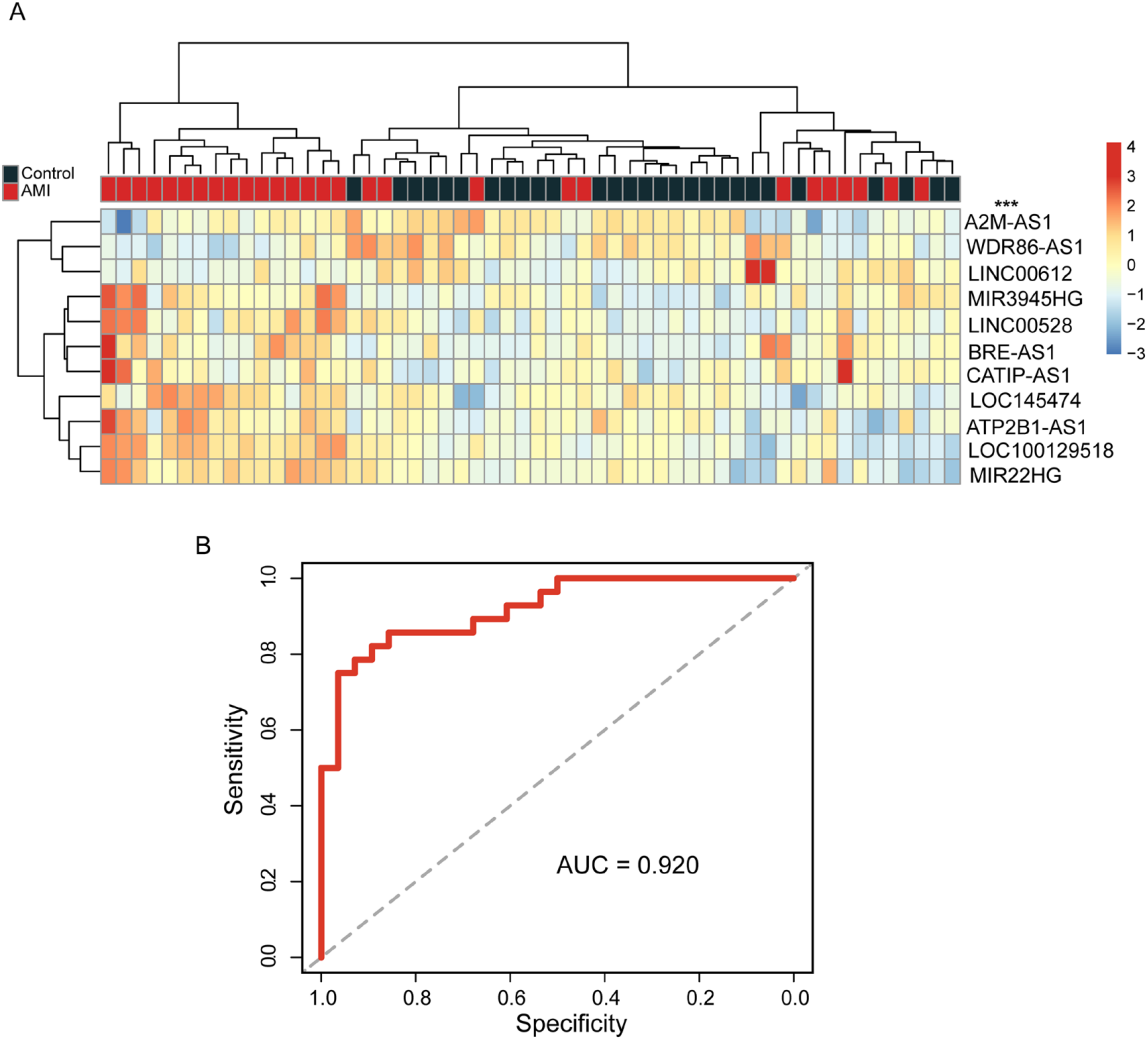
Validation of candidate lncRNA biomarkers in distinguishing between AMI patients and healthy samples in the internal validation cohort **(A)** The heatmap of hierarchical clustering of candidate lncRNA biomarkers for all samples in the internal validation cohort. **(B)** Receiver operating characteristic curves of SVM-based lncRNA risk classifier in the internal validation cohort.

We validated the predictive value of these 11 candidate lncRNA biomarkers in the internal validation cohort. Hierarchical clustering analysis of all samples in the internal validation cohort also reveals two distinctive sample groups: the first sample group contained 16 AMI samples and the second sample group contained all healthy samples (n=28) and 12 of 28 AMI patients achieving a prediction accuracy of 78.6% (Figure 2A). The disease status of the two sample groups was significantly different (p<0.001, chi-square test). Then SVM-based lncRNA risk classifier was tested in the internal validation cohort using LOOCV strategy. The ROC curves of lncRNA risk classifier reflected separation between AMI and healthy samples, with an AUC of 0.92, the sensitivity of 78.6% and specificity of 89.3% (Figure 2B). The DOR was 30.6.

### Further validation of candidate lncRNA biomarkers in the independent validation cohort

**Figure 3:**
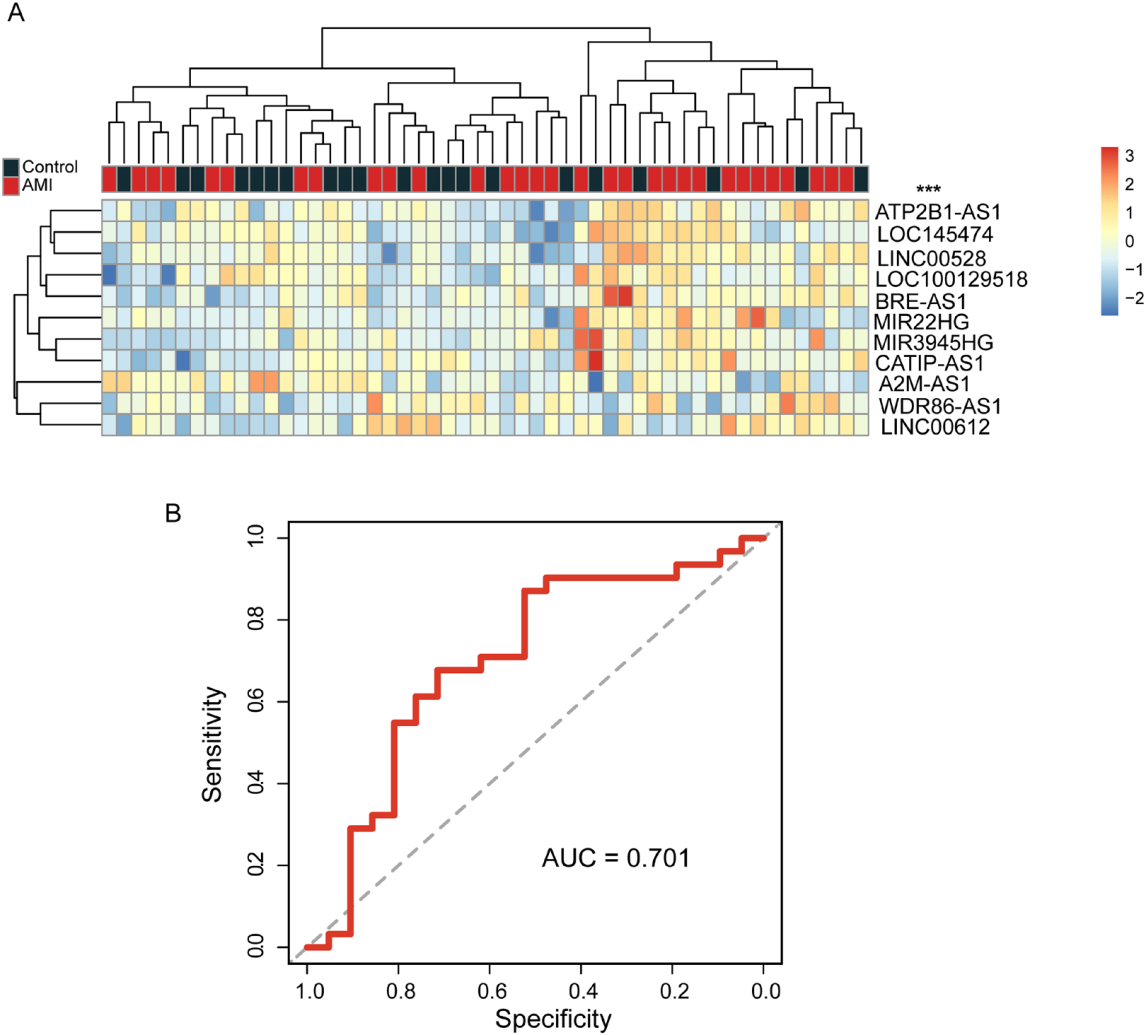
Further evaluation of candidate lncRNA biomarkers for early diagnosis of AMI in the independent validation cohort **(A)** The heatmap of hierarchical clustering of candidate lncRNA biomarkers for all samples in the independent validation cohort. **(B)** Receiver operating characteristic curves of SVM-based lncRNA risk classifier in the independent validation cohort.

Further validation of the predictive value of candidate lncRNA biomarkers in early diagnosis of AMI was conducted using another independent validation cohort. Hierarchical clustering of all samples (n=52) also revealed clear distinctions between AMI patients and healthy samples: the first sample group contained 32 samples (16 AMI patients and 16 healthy samples) and the second sample group contained 20 samples (15 AMI patients and 5 healthy samples) achieving a prediction accuracy of 61.5% (Figure 3A). The disease status of the two sample groups was marginally significantly different (p=0.08, chi-square test). When the SVM-based lncRNA risk classifier was applied to evaluate the risk of AMI and healthy samples, it performed remarkably well. The discriminatory power measured by the AUC and DOR was 0.701 and 7 (Figure 3B), respectively. Sensitivity and specificity were 90.3% and 42.9%, respectively.

### Identification of recurrence-related lncRNAs in AMI

**Figure 4:**
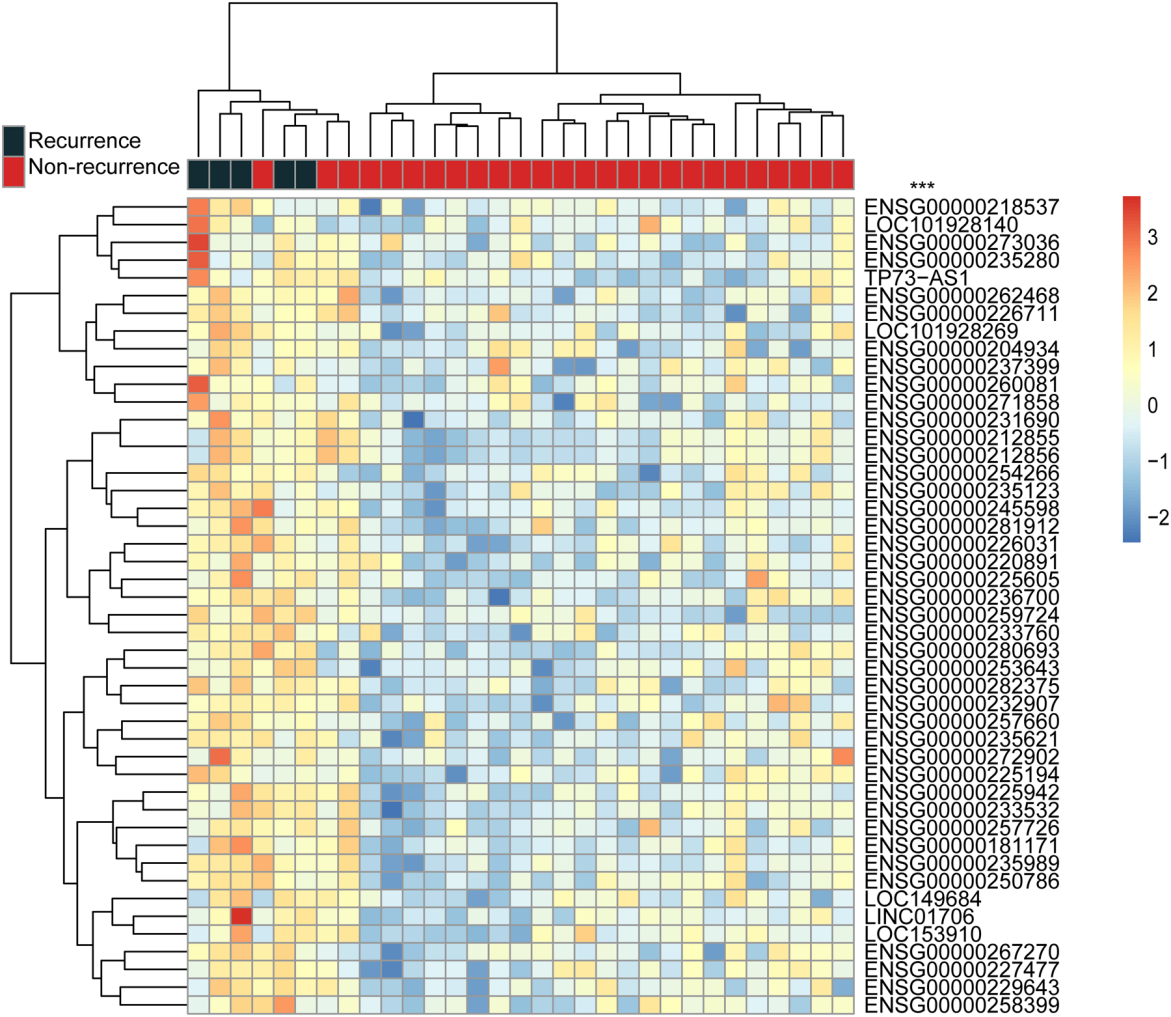
Unsupervised hierarchical clustering of AMI patients with and without recurrent events based on expression levels of 46 significantly differentially expressed lncRNAs

To identify potential lncRNAs involved in disease recurrence in AMI, we also performed SAM analysis and identified 46 lncRNAs which were significantly differentially expressed (p-value <0.05 after Benjamini & Hochberg correction) in AMI patients with recurrent events (n=5) versus AMI patients without any recurrent events (n=26). All the 46 lncRNAs were unregulated in AMI patients with recurrent events (Supplementary Table 2). Figure 4 illustrates the results of hierarchical clustering of all samples (n=31) representing two distinctive sample groups: the first sample group contained all samples with recurrent events and 2 of 26 AMI patients without any recurrent events and the second sample group contained 23 patients without any recurrent events achieving a prediction accuracy of 90.3%. The recurrence status of the two patient groups was significantly different (p<0.001, chi-square test).

### Functional analysis of candidate lncRNA biomarkers

To understand the functional roles of candidate lncRNA biomarkers, we first examined the expression correlation by Pearson correlation coefficients between protein-coding genes (PCGs) expression and lncRNA expression in the discovery cohort and identified 1969 PCGs that are co-expressed with at least one of 11 candidate lncRNA biomarkers as previously described. Then we used DAVID for functional enrichment analysis for PCGs co-expressed with candidate lncRNA biomarkers to infer the functional roles of candidate lncRNA biomarkers. Functional classification of these 1969 PCGs co-expressed with candidate lncRNA biomarkers and a statistical cut-off criterion of FDR-adjusted p-value of 0.05 indicated significant enrichment of these genes in 11 GO-BP terms (Table 1) and 9 KEGG pathways (Table 2). From these tables, it can be observed that the majority of enriched GO and KEGG functional categories were found to be associated with immune-related biological processes involved in AMI.

**Table 1:** Significantly enriched gene ontology (GO) terms

GO Term	Total number of genes	Fold enrichment	FDR
GO:0006954∼inflammatory response	72	3.149	8.19E-15
GO:0071222∼cellular response to lipopolysaccharide	26	3.814	2.06E-05
GO:0006955∼immune response	56	2.205	8.84E-05
GO:0031663∼lipopolysaccharide-mediated signaling pathway	13	6.734	2.91E-04
GO:0045087∼innate immune response	55	2.120	4.31E-04
GO:0030593∼neutrophil chemotaxis	18	4.521	4.47E-04
GO:0006935∼chemotaxis	23	3.125	6.22E-03
GO:0050900∼leukocyte migration	23	3.125	6.22 E-03
GO:0032729∼positive regulation of interferon-gamma production	13	4.685	0.023
GO:0032088∼negative regulation of NF-kappaB transcription factor activity	16	3.736	0.032
GO:0007166∼cell surface receptor signaling pathway	36	2.178	0.039

**Table 2:** Significantly enriched KEGG pathways

Pathway name	Total number of genes	Fold enrichment	FDR
hsa04380:Osteoclast differentiation	35	3.996	3.01E-09
hsa05150:Staphylococcus aureus infection	19	5.263	6.66E-06
hsa05323:Rheumatoid arthritis	23	3.909	5.29E-05
hsa05152:Tuberculosis	34	2.873	6.10E-05
hsa05140:Leishmaniasis	20	4.213	1.40E-04
hsa04145:Phagosome	28	2.737	0.003
hsa04064:NF-kappa B signaling pathway	20	3.438	0.004
hsa04060:Cytokine-cytokine receptor interaction	35	2.276	0.01
hsa04668:TNF signaling pathway	21	2.963	0.024

## DISCUSSION

Myocardial infarction remains a leading cause of death and disability worldwide despite substantial improvements in diagnosis over the past decade. Early detection of AMI is extremely important for the effective management of patients and selects appropriate treatment. Although traditionally available biomarkers, such as Troponin (cTnI/T) cardiac troponins (cTns) and creatine kinase-MB (CK-MB) have greatly enabled the clinicians in the rapid diagnosis, they suffer from a lack of high specificity. Recent genetic studies have demonstrated the molecular heterogeneity of AMI suggesting the genetic component has an important role in the development of AMI.

During past years, lncRNAs have been found to be an important genetic component involved in genome regulatory network and play important roles in disease processes [6]. Transcriptome research shows that lncRNAs have high tissue- and cell type-specific expression patterns, implying their importance as biomarkers in diagnosis and prognosis in diseases [4]. Increasing efforts have been made for identifying diagnostic and prognostic lncRNA biomarkers in various human cancers [14–19], there is a lack of the investigation into the utility of lncRNAs as biomarkers for early diagnosis of AMI. Several profile-based studies have identified altered lncRNA expression involved in the initiation and progression of MI. Zangrando *et al.* used microarray analysis of 4 MI and 4 sham-operated mice sacrificed 24 hours after surgery to investigate the role of lncRNAs in left ventricular remodeling and identified 30 differentially expressed lncRNAs [11]. Another study performed by Qu and colleagues identified 545 deregulated lncRNAs involved in cardiac fibrogenesis induced by MI using microarray analysis [20]. Recently two studies constructed dysregulated lncRNA-mRNA co-expressed network to investigate the functional roles of lncRNAs in MI and identified some candidate key lncRNAs in MI [21, 22], emphasizing the potential of lncRNAs as biomarkers for early diagnosis of AMI.

To meet this need, in the present study, we obtained lncRNA expression profiles on two cohorts of 151 samples from the Gene Expression Omnibus (GEO) by repurposing microarray data and compared lncRNA expression profiles between AMI patients and healthy samples. By comparing the expression levels of lncRNAs between AMI patients and healthy samples, we found that 11 lncRNAs are differentially expressed in AMI compared with healthy samples, indicating that lncRNAs may have critical roles in the occurrence of AMI. Such differentiation signified their diagnostic roles as biomarkers to distinguish between AMI patients and healthy samples. By using hierarchical clustering analysis and an SVM algorithm, the predictive power of these 11 differential lncRNA biomarkers in distinguishing between AMI patients and healthy samples was validated in the discovery cohort and two independent patient cohorts. Although some of differential lncRNA biomarkers have been reported to be aberrantly expressed in cancers, diagnostic roles of these differential lncRNA biomarkers have not been studied in AMI. For example, BRE-AS1 has been reported to be differentially regulated in NSCLC tumors [23]. Long ncRNA MIR22HG could repressed hepatocellular carcinoma cell invasion by deriving miR-22 and targeting HMGB1 [24]. Long ncRNA MIR3945HG has been identified as novel candidate diagnostic markers for tuberculosis [25]. The functional roles of remaining 8 out of 11 differential lncRNA biomarkers are still unknown.

Previous studies have found that lncRNAs and their co-expressed PCGs tended to be involved in the same biological process. Therefore, it is possible to infer lncRNA function by associating specific lncRNAs with biological processes of their co-expressed PCGs [10, 26]. Here, in order to predict the putative function of 11 differential lncRNA biomarkers in AMI, we performed co-expressed analysis for lncRNAs with protein-coding genes and identified some PCGs that have a common expression pattern of 11 differential lncRNA biomarkers. Then GO and KEGG enrichment analysis was used to associate specific lncRNAs with biological processes. The results of enrichment analysis suggested that these 11 differential lncRNA biomarkers were enriched in important biological processes involved in AMI. For example, inflammation- and immune-related biological processes have been reported to play an essential role in cardiac injury and repair, and together with the activation of innate and adaptive immune responses have been proven to be the hallmark of MI [27, 28]. Genetic variations in the receptor for lipopolysaccharides have been found to be a risk factor for MI and Lipopolysaccharide pretreatment attenuates myocardial infarct size [29, 30]. NFkB is an important transcription factor involved in many cell survival pathways and Santos found that polymorphism in NFkB is associated with heart function in patients with heart failure [31].

Some limitations of our study should be acknowledged. Firstly, lncRNA number included in this study was relatively less compared to known lncRNAs in some databases because lncRNA expression profiles were obtained based on HG-U133 Plus 2.0 arrays. Secondly, recurrence-related lncRNAs identified in this study was not validated in the independent patient cohort due to the limitation of the available patient dataset with the recurrent event. Finally, further experimental verification should be carried out to study the functional roles of these candidate lncRNA biomarkers in AMI which will improve our understanding of molecular mechanism of the occurrence and recurrence of AMI.

## MATERIALS AND METHODS

### Datasets

The following two independent cohorts of AMI patient and their gene expression data were obtained from the publicly available Gene Expression Omnibus (*GEO*) database (www.ncbi.nlm.nih.gov/geo/) and were included in our study: GSE66360 (https://www.ncbi.nlm.nih.gov/geo/query/acc.cgi?acc=GSE66360) contained 49 AMI patients and 50 healthy samples. GSE48060 (https://www.ncbi.nlm.nih.gov/geo/query/acc.cgi?acc=GSE48060) contained 31 AMI patients and 21 healthy samples as well as 5 AMI patients with recurrent events and 26 AMI patients without any recurrent events over an 18-month follow-up. Among two data patient cohorts, the dataset GSE66360 was divided into the discovery cohort composing of 21 AMI patients and 22 healthy samples and internal validation cohort composing of 28 AMI patients and 28 healthy samples according to the classification information in the original experiment. The GSE48060 was used as an independent validation cohort.

### Acquisition and processing of lncRNA expression profiles

The raw CEL files were downloaded from GEO database and background correction, quantile normalization and log2-transformation using Robust Multichip Average (RMA) method. The lncRNA expression data in two patient cohorts were obtained by re-annotating probes strategy according to previous studies [17, 32]. Briefly, based on NetAffx Annotation Files (HG-U133 Plus 2.0 Annotations), probe sets were mapped to RefSeq transcript ID and/ or Ensembl gene ID. Only probe sets mapping to lncRNA annotation from GENCODE were retained which resulting in 2466 annotated lncRNA genes.

### Statistical analysis

Significance analysis of microarrays SAM method was used to identify differentially expressed lncRNAs between AMI patients and healthy samples with an adjusted P-value <0.05 after Benjamini & Hochberg correction and fold change > 2.0 (<0.5). Hierarchical clustering of the expression values of differentially expressed lncRNAs was performed with R software using the metric of euclidean distance and complete linkage. The chi-square test was used to evaluate the significance of the association between lncRNA expression pattern and disease status. The support vector machine (SVM) with the sigmoid kernel method was used to develop a lncRNA risk classifier and the performance was estimated using the leave one out cross-validation (LOOCV). After that, a receiver operating characteristic (ROC) curve analysis was carried out with lncRNA risk classifier distinguishing between AMI patients and healthy samples, and the area under ROC curve (AUC) was computed to estimate the diagnostic accuracy of lncRNA risk classifier. The diagnostic odds ratio (DOR) was calculated to evaluate the diagnostic value of lncRNA risk classifier.

### Function enrichment analysis

The Database for Annotation, Visualization and Integrated Discovery (DAVID, v6.8, https://david.ncifcrf.gov/home.jsp) was used for functional enrichment analysis for PCGs. The functional enrichment analysis was limited in GO-biological processes and KEGG-PATHWAY. Only those terms or pathways that reported an FDR-adjusted p-value of 0.05 were selected as significantly enriched functional annotations.

## SUPPLEMENTARY MATERIALS TABLES


