# Supplementary material for: Differential expression profiles of long non-coding RNAs as potential biomarkers for the early diagnosis of acute myocardial infarction
Source: Oncotarget. 2017 Aug 9;8(51):88613–21. doi: 10.18632/oncotarget.20101 (PMC5687631; doi:10.18632/oncotarget.20101)
Supplement: Supplementary file 1 [file oncotarget-08-88613-s001.pdf]

Differential expression profiles of long non-coding RNAs as potential biomarkers for the early diagnosis of acute myocardial infarction

SUPPLEMENTARY MATERIALS

Supplementary Table 1: Overview of differentially expressed lncRNAs between AMI patients and healthy samples

Ensembl ID	Gene Name	Location	Fold Change	q-value(%)
LOC145474		Chromosome 14: 71,487,861-71,489,714 forward strand	2.264	0
LOC100129518		Chromosome 6: 159,760,258-159,762,332 reverse strand	3.489	0
BRE-AS1		Chromosome 2: 27,889,456-27,891,114 reverse strand	2.588	0
ENSG00000186594	MIR22HG	Chromosome 17: 1,711,493-1,717,174 reverse strand.	2.59	0
ENSG00000251230	MIR3945HG	Chromosome 4: 184,844,585-184,855,751 reverse strand	3.038	0
ENSG00000271614	ATP2B1-AS1	Chromosome 12: 89,708,959-89,712,590 forward strand	2.539	0
ENSG00000225062	CATIP-AS1	Chromosome 2: 218,366,665-218,367,835 reverse strand.	2.066	0
ENSG00000269220	LINC00528	Chromosome 22: 17,777,322-17,779,481 forward strand	2.333	0
ENSG00000243836	WDR86-AS1	Chromosome 7: 151,409,161-151,413,354 forward strand	0.472	0
ENSG00000245105	A2M-AS1	Chromosome 12: 9,065,177-9,068,060 forward strand	0.474	0
ENSG00000214851	LINC00612	Chromosome 12: 9,055,586-9,065,070 reverse strand	0.468	0

Supplementary Table 2: Overview of differentially expressed lncRNAs between AMI patients with recurrent events and those without any recurrent events

Gene id	Fold Change	q-value(%)
LINC01706	1.3	0
ENSG00000282375	1.211	0
LOC101928269	1.241	0
ENSG00000229643	1.224	0
ENSG00000218537	1.255	0
ENSG00000225605	1.166	0
ENSG00000262468	1.245	0
ENSG00000257660	1.205	0
ENSG00000235280	1.176	0
ENSG00000259724	1.134	0
ENSG00000233760	1.16	0
ENSG00000235989	1.19	0
LOC101928140	1.205	0
ENSG00000280693	1.203	0
ENSG00000226031	1.22	0
ENSG00000253643	1.125	0
ENSG00000272902	1.163	0
ENSG00000236700	1.171	0
ENSG00000267270	1.106	0
ENSG00000250786	1.202	0
LOC153910	1.147	0
ENSG00000235621	1.186	0
ENSG00000273036	1.161	0
ENSG00000225942	1.143	0
ENSG00000204934	1.181	0
ENSG00000181171	1.202	0
ENSG00000231690	1.172	0
ENSG00000258399	1.157	0
ENSG00000233532	1.151	0
ENSG00000227477	1.159	0
ENSG00000257726	1.171	0
ENSG00000226711	1.092	0
ENSG00000245598	1.144	0
ENSG00000220891	1.127	0
ENSG00000281912	1.147	0
ENSG00000225194	1.147	0
LOC149684	1.103	0
ENSG00000232907	1.172	0
ENSG00000260081	1.161	0
ENSG00000212855	1.163	0
ENSG00000212856	1.163	0
TP73-AS1	1.098	0
ENSG00000254266	1.089	0
ENSG00000271858	1.151	0
ENSG00000237399	1.149	0
ENSG00000235123	1.188	0
